# The Effectiveness of Digital Health Lifestyle Interventions on People With Prediabetes: Protocol for a Systematic Review, Meta-Analysis, and Meta-Regression

**DOI:** 10.2196/50340

**Published:** 2024-02-09

**Authors:** Tanja Fredensborg Holm, Flemming Witt Udsen, Kristine Færch, Morten Hasselstrøm Jensen, Bernt Johan von Scholten, Ole Kristian Hejlesen, Stine Hangaard

**Affiliations:** 1 Department of Health Science and Technology Aalborg University Gistrup Denmark; 2 Steno Diabetes Center North Jutland Aalborg University Hospital Aalborg Denmark; 3 Data Science Novo Nordisk A/S Søborg Denmark; 4 Clinical Research Copenhagen University Hospital – Steno Diabetes Center Copenhagen Herlev Denmark

**Keywords:** digital health, effectiveness, lifestyle intervention, meta-analysis, meta-regression, prediabetic state, systematic review, type 2 diabetes prevention, weight loss

## Abstract

**Background:**

There has been an increasing interest in the use of digital health lifestyle interventions for people with prediabetes, as these interventions may offer a scalable approach to preventing type 2 diabetes. Previous systematic reviews on digital health lifestyle interventions for people with prediabetes had limitations, such as a narrow focus on certain types of interventions, a lack of statistical pooling, and no broader subgroup analysis of intervention characteristics. The identified limitations observed in previous systematic reviews substantiate the necessity of conducting a comprehensive review to address these gaps within the field. This will enable a comprehensive understanding of the effectiveness of digital health lifestyle interventions for people with prediabetes.

**Objective:**

The objective of this systematic review, meta-analysis, and meta-regression is to systematically investigate the effectiveness of digital health lifestyle interventions on prediabetes-related outcomes in comparison with any comparator without a digital component among adults with prediabetes.

**Methods:**

This systematic review will include randomized controlled trials that investigate the effectiveness of digital health lifestyle interventions on adults (aged 18 years or older) with prediabetes and compare the digital interventions with nondigital interventions. The primary outcome will be change in body weight (kg). Secondary outcomes include, among others, change in glycemic status, markers of cardiometabolic health, feasibility outcomes, and incidence of type 2 diabetes. Embase, PubMed, CINAHL, and CENTRAL (Cochrane Central Register of Controlled Trials) will be systematically searched. The data items to be extracted include study characteristics, participant characteristics, intervention characteristics, and relevant outcomes. To estimate the overall effect size, a meta-analysis will be conducted using the mean difference. Additionally, if feasible, meta-regression on study, intervention, and participant characteristics will be performed. The Cochrane risk of bias tool will be applied to assess study quality, and the GRADE (Grading of Recommendations Assessment, Development and Evaluation) approach will be used to assess the certainty of evidence.

**Results:**

The results are projected to yield an overall estimate of the effectiveness of digital health lifestyle interventions on adults with prediabetes and elucidate the characteristics that contribute to their effectiveness.

**Conclusions:**

The insights gained from this study may help clarify the potential of digital health lifestyle interventions for people with prediabetes and guide the decision-making regarding future intervention components.

**Trial Registration:**

PROSPERO CRD42023426919; http://tinyurl.com/d3enrw9j

**International Registered Report Identifier (IRRID):**

PRR1-10.2196/50340

## Introduction

Diabetes is a major global health care challenge. In 2021, an estimated 10.5% of the world’s population aged between 20 and 79 years had diabetes, and the prevalence is expected to increase to 12.2% by 2045 [1]. Type 2 diabetes (T2D) is the predominant form of diabetes, representing approximately 90% of diabetes cases worldwide, and is one of the most common metabolic disorders [1,2]. T2D results in abnormally high levels of plasma glucose due to insulin resistance and gradual impairment of pancreatic beta-cell function [2,3]. Continuously high levels of plasma glucose can lead to several micro- and macrovascular complications, such as retinopathy, nephropathy, and cardiovascular diseases, increasing the risk of premature mortality, morbidity, and reduced quality of life [2,3]. This poses a major economic burden to the health care system and society [3-5].

The development of T2D is affected by both environmental and genetic factors but living with overweight and having a sedentary lifestyle are prominent risk factors [2,6]. Before the diagnosis of T2D, an intermediate stage of elevated plasma glucose usually exists [7,8]. This intermediate stage, where a person has abnormally high glucose levels but not high enough to be diagnosed with T2D, is called prediabetes [1,9]. Prediabetes is characterized by impaired fasting glucose (IFG), impaired glucose tolerance (IGT), or elevated hemoglobin A_1c_ (HbA_1c_) levels [9,10]. Depending on the organization, diagnostic test, and cutoff criteria used, different definitions of prediabetes exist, which makes global prevalence estimates challenging and varying [8,11]. According to the International Diabetes Federation, an estimated 10.6% of the global population aged between 20 and 79 years had IGT in 2021 and an estimated 6.2% of adults had IFG, based on the criteria established by the World Health Organization. The global prevalence of IGT and IFG is expected to increase to 11.4% and 6.9%, respectively, by 2045 [1].

Most people with prediabetes are unaware of the condition, as it is frequently asymptomatic [10]. People with prediabetes are at increased risk of developing T2D. Approximately 70% of people with prediabetes will develop T2D during their lifetime and about 25% will progress to T2D within 3-5 years [10,11]. Additionally, elevated plasma glucose in the range of prediabetes leads to an increased risk of developing micro- and macrovascular complications usually associated with T2D [12-14]. Several studies have demonstrated that progression from prediabetes (mainly IGT) to T2D, as well as complications, can be prevented and delayed through intensive lifestyle interventions that focus on diet and physical activity [15-18]. These studies have found that intensive lifestyle interventions reduced the risk of progression to T2D by 28%-58% over a 2.5- to 6-year period [15-18]. Prediabetes thus represents a time of opportunity to prevent or delay progression to T2D, potentially slowing down the increase in the prevalence of people with T2D [10,11,19].

Despite the effect of resource-intensive lifestyle interventions observed in clinical trials, these interventions may not be scalable and accessible in clinical practice because of restricted health care resources [8,20,21]. Digital health interventions have the potential to reduce the resources of intensive lifestyle interventions and thereby make them more scalable and accessible in clinical practice [21-23]. Digital health defines the use of digital technologies in support of health care and includes technologies such as mobile apps, videos, websites, and wearable devices [24,25]. Previous reviews have demonstrated that digital health interventions are effective in reducing body weight among people with overweight or obesity [26,27] and reducing HbA_1c_ among people with diabetes [28-30]. These findings indicate that digital health interventions have the potential to improve various health-related outcomes. However, more research is still required to determine whether digital health lifestyle interventions can effectively improve prediabetes-related outcomes (eg, weight loss and glycemic status) to clarify their potential for people with prediabetes. In addition, an evaluation of characteristics associated with effects on outcomes is crucial to guide the development of future interventions and inform decision-making on intervention components.

Previous systematic reviews have investigated the effect of digital health lifestyle interventions on people with prediabetes [20,21,31-33]. However, the reviews had limitations. They focused on specific types of digital interventions [32], including a relatively small number of data sources [32,33], lacking a meta-analysis [20,21,33], or pooling results from different study designs [31]. Furthermore, previous meta-analyses did not include a broader subgroup analysis exploring the association between intervention characteristics and effects on outcomes [31,32]. As a result, the specific characteristics that are associated with effects on prediabetes-related outcomes remain relatively unknown. Additionally, given the rapid development of digital interventions, several new research papers have likely emerged since the conduct of previous reviews.

The considerations mentioned above indicate the need for an updated and comprehensive systematic review, meta-analysis, and meta-regression in the field to synthesize and evaluate the effectiveness of digital health lifestyle interventions on people with prediabetes.

## Methods

### Study Design

The conduct and reporting of this review will follow the PRISMA (Preferred Reporting Items for Systematic Reviews and Meta-Analyses) guidelines [34]. The review process will be conducted based on this review protocol, which adheres to the PRISMA-P (Preferred Reporting Items for Systematic Review and Meta-Analysis Protocols) checklist [35]. The protocol was registered on PROSPERO (International Prospective Register of Systematic Reviews) on July 5, 2023 (CRD42023426919), in accordance with the PRISMA guidelines.

### Objective

The objective of this systematic review, meta-analysis, and meta-regression is to systematically investigate the effectiveness of digital health lifestyle interventions on weight loss and other prediabetes-related secondary outcomes in comparison with any comparator without a digital component among adults with prediabetes.

### Review Questions

This systematic review, meta-analysis, and meta-regression is guided by the following research questions: What is the effectiveness of digital health lifestyle interventions on weight loss and other prediabetes-related outcomes among adults with prediabetes, and how do variations across study, intervention, and participant characteristics contribute to differences in the observed effect?

### Inclusion Criteria

#### Participants

Studies including adults aged 18 years or older meeting criteria indicative of prediabetes will be considered for inclusion in this systematic review, meta-analysis, and meta-regression. The criteria for meeting prediabetes will be defined by the presence of IFG, IGT, or raised HbA_1c_. According to the criteria of the American Diabetes Association, IFG is defined as fasting plasma glucose levels between 5.6-6.9 mmol/L, IGT as 2-hour plasma glucose (75-gram oral glucose tolerance test) between 7.8-11.0 mmol/L, and raised HbA_1c_ as HbA_1c_ levels between 39-47 mmol/mol [9].

Studies that are restricted to adults with T2D, type 1 diabetes, or gestational diabetes will be excluded. In addition, studies that investigate the effect of a combined lifestyle and pharmacological intervention will be excluded. Studies that examine combined age groups (eg, adults and adolescents) will also be excluded unless the data for the adult population are reported transparently and separately. Similarly, studies that do not report separate data when considering prediabetes in conjunction with any type of diabetes (eg, T2D) or metabolic syndrome will also be excluded.

#### Interventions

Studies that examined the effectiveness of digital health lifestyle interventions (diet or physical activity interventions) on adults with prediabetes will be considered for inclusion. These studies may encompass both independent digital solutions and interventions that combine in-person meetings with a digital component. The digital component may include different technologies such as SMS text messaging, smartphone apps, email, automated phone calls, mobile phones, websites, computer-based programs, and video. Studies will be deemed ineligible for inclusion if the intervention does not incorporate a digital component.

#### Comparators

This systematic review, meta-analysis, and meta-regression will examine studies that compare digital health lifestyle interventions to any comparator without a digital component.

#### Settings

Studies investigating the effectiveness of digital health lifestyle interventions on adults with prediabetes will be considered without any restrictions on the setting.

#### Outcomes

Studies that report the effectiveness on relevant outcomes related to prediabetes (eg, weight loss, glycemic status, and incidence of T2D) will be considered in this systematic review, meta-analysis, and meta-regression. The primary outcome will be a change in body weight (kg). Secondary outcomes will include changes in the following variables: (1) glycemic status, (2) body composition, (3) incidence of T2D, (4) markers of cardiometabolic health (eg, blood pressure and lipids), (5) patient-reported outcomes (eg, quality of life), and (6) feasibility outcomes (eg, differential retention rate, adherence, and acceptance).

#### Study Types

Randomized controlled trials with a parallel design will be eligible for inclusion in the systematic review, meta-analysis, and meta-regression. Additionally, studies will be eligible for inclusion if the researchers assess that the study was conducted using a parallel randomized controlled trial design, regardless of whether the paper itself describes the design using a different terminology. Systematic reviews or meta-analyses will be excluded but inspected for potentially eligible studies. Only full-text studies that have undergone peer review and are available in English, Norwegian, Danish, or Swedish will be included. The present review will not impose any restrictions on the publication year of included studies, as older as well as more recent interventions will be evaluated to offer valuable insights.

### Search Strategy

The systematic search will be conducted by the first author (TFH) across various databases with the aim of systematically identifying studies investigating the effectiveness of digital health lifestyle interventions for adults with prediabetes. The following bibliographic databases will be searched: Embase, PubMed, CINAHL, and CENTRAL (Cochrane Central Register of Controlled Trials). Assistance in the performance of the systematic search will be provided by a research librarian experienced in systematic review searching.

To identify search terms of relevance, encompassing controlled vocabulary and keywords, an initial limited search will be conducted in PubMed and Embase. The systematic search will involve the identification and incorporation of related terms and synonyms associated with the identified search terms. Additionally, a series of search functions, such as truncation and phrase search, will be used, and the identified search terms will be combined using Boolean operators. The systematic search approach will be tailored and adjusted to suit each database included in the study. The complete search strategy for PubMed is presented in the Multimedia Appendix 1. If relevant, for example, in case of inaccessibility of a study or questions during the selection process, the authors of the study in question will be contacted.

Reference search and citation tracking of eligible studies will be performed to identify additional potentially relevant studies. Citation tracking will be conducted in Web of Science and SCOPUS.

A follow-up search will be conducted in each included database before final submission to prevent selection bias and to ensure that all newly published papers are included.

### Study Selection

The identified studies will be collected and imported into RefWorks. First, any duplicate studies will be removed in RefWorks. Second, the titles and abstracts of remaining studies will be screened for inclusion against the review eligibility criteria by the first author. Third, the studies that meet eligibility criteria, as well as those where uncertainty persists, will undergo full-text screening. The first author will assess their inclusion against the predetermined eligibility criteria with assistance from a coauthor. In case of any disagreement between the reviewers regarding study selection, the reviewers will engage in discussion to resolve the issue. If necessary, a third coauthor will be involved to reach a consensus. Data extraction, quality assessment, and, if feasible, statistical analysis will be conducted for all eligible studies.

A PRISMA flowchart [34] will be used to demonstrate the screening process for each stage and the results of the systematic search, as depicted in the Multimedia Appendix 2. The PRISMA flowchart will also comprise exclusion reasons of full-text studies.

### Risk of Bias Assessment

The Cochrane risk of bias tool will be applied to facilitate the quality assessment of the included studies by the first author with assistance from a coauthor. In case of disagreements, the reviewers will resolve them through discussion, and a third coauthor may be involved if needed. If necessary, for example, if items are missing or unclear for the risk of bias assessment, the authors of the studies in question will be contacted.

### Data Extraction

A standardized spreadsheet in Excel (Microsoft Corporation) will be used to extract data from included studies, facilitating data synthesis and analysis while ensuring consistent data extraction. The first author will perform the data extraction with assistance from a coauthor. In case of any disagreement between the reviewers regarding data extraction, the reviewers will engage in discussion to resolve the issue. If necessary, a third coauthor will be involved to reach a consensus.

The data items that will be extracted include the following four categories:

1. Study characteristics, including first author, year of publication, study design, and country.
2. Baseline characteristics of study participants, including sample size of each group, percentage male participants, ethnicity, age, body weight, BMI, HbA_1c_, and fasting plasma glucose.
3. Outcomes of the study intervention and control group, including primary and/or secondary outcomes when available.
4. Characteristics of the digital health lifestyle intervention, including duration of follow-up, setting, resources used, intervention components, mode of delivery, provider, contact type (eg, face-to-face, fully automated, or remote), and frequency of contact.

### Data Synthesis

A statistical meta-analysis will be conducted using Stata (version 17; StataCorp 2021) by pooling data from included studies to estimate the overall effect size of digital health lifestyle interventions on adults with prediabetes. The mean difference between the digital intervention group and the comparator group will be used to present the effect size for continuous data, along with a 95% CI. Primary outcomes presented in other units will be scaled to the same unit if possible (eg, a change in body weight reported as percentage or in pounds will be transformed into kilograms). Standardized mean difference will be used to present the effect size for secondary outcomes presented in different units unless it is possible to scale them to the same unit. Traditional methods will be used to convert results reported as medians and interquartile ranges into means and SDs [36]. For dichotomous data, the effect size will be presented as an odds ratio with a 95% CI. In cases where statistical pooling is impossible, the results will be presented narratively, accompanied by tables and figures where applicable. Feasibility outcomes will also be summarized narratively, eventually supported by tables and figures.

Both qualitative and statistical assessments will be conducted to evaluate the heterogeneity of the included studies. Qualitative assessment will involve comparing the characteristics of the studies, while the *I*^2^ test will be used for statistical analysis. If heterogeneity is present, as determined by an *I*^2^ value of more than 50%, a random-effects model will be used to statistically pool the data. Otherwise, a fixed-effect model will be used. The results of the meta-analysis will be presented in a forest plot. If possible, meta-regression on study characteristics (eg, variations across countries), intervention characteristics (eg, human-to-human interventions vs fully automated interventions), and participant characteristics (eg, gender) will be conducted to uncover common patterns among effective interventions and identify knowledge gaps within the research field. If conducting a meta-regression is impossible due to data limitations, alternative subgroup analyses based on study-, intervention-, and participant characteristics will be performed.

To visually evaluate the potential presence of publication bias, a funnel plot will be created and included in the presentation of the results. If the number of studies included in the meta-analysis is more than 10, a statistical test (Egger test) will be performed to detect funnel plot asymmetry.

### Certainty of Evidence Assessment

The certainty of evidence will be assessed using the GRADE (Grading of Recommendations Assessment, Development and Evaluation) approach [37]. The assessment will consider the risk of bias, inconsistency, indirectness, imprecision, and publication bias. Based on these considerations, the evidence will be classified as high, moderate, low, or very low certainty. A summary of findings table will be generated using the web-based software GRADEpro Guideline Development Tool to summarize the strength and reliability of the evidence [38].

## Results

The results of this review intend to provide insights into both the overall effectiveness of digital health lifestyle interventions and the characteristics that contribute to their effectiveness. The results will undergo peer review and be submitted for publication.

## Discussion

This systematic review, meta-analysis, and meta-regression will review current evidence to address a lack of comprehensive research within the field of digital health lifestyle interventions for people with prediabetes.

As this publication represents a preliminary stage of the systematic review, specific comparisons with key studies are currently lacking. However, the protocol emphasizes conducting a comprehensive review of relevant literature to allow for meaningful comparisons once the results are obtained. Furthermore, the forthcoming discussion aims to assess the applicability of the findings in clinical practice and their potential implications for real-world implementation. Evaluating the resources used in the included interventions may be crucial for this assessment, considering their potential limitations in expanding digital health lifestyle interventions into real-world settings.

The insight gained from this study may help clarify the potential of digital health lifestyle interventions in the management of prediabetes and offer guidance for future development decisions. The planned systematic evidence assessment, using the GRADE methodology, is expected to indirectly inform and guide future recommendations and development decisions for digital health solutions [39]. These recommendations will be enriched by insights obtained from the meta-regression or subgroup analysis. We intend to explore these aspects in the forthcoming discussion.

However, it is crucial to acknowledge the anticipated limitations within this review. Studies investigating digital health interventions are expected to vary in factors such as mode of delivery, intervention type, population, and duration, resulting in notable heterogeneity among the included studies. This diversity might complicate the comparison and statistical pooling of results. Moreover, the upcoming discussion intends to highlight additional limitations and knowledge gaps identified during the review, with the aim of informing and enhancing further work in this field.

## Appendix

Multimedia Appendix 1 Search strategy.

Multimedia Appendix 2 Study selection process.

## Abbreviations

A/S: joint-stock company, in Danish
CENTRAL: Cochrane Central Register of Controlled Trials
GRADE: Grading of Recommendations Assessment, Development and Evaluation
HbA_1c_: hemoglobin A_1c_
IFG: impaired fasting glucose
IGT: impaired glucose tolerance
PRISMA: Preferred Reporting Items for Systematic Reviews and Meta-Analyses
PRISMA-P: Preferred Reporting Items for Systematic Review and Meta-Analysis Protocols
PROSPERO: International Prospective Register of Systematic Reviews
T2D: type 2 diabetes
