# Supplementary material for: The Effectiveness of Digital Health Lifestyle Interventions on People With Prediabetes: Protocol for a Systematic Review, Meta-Analysis, and Meta-Regression
Source: JMIR Res Protoc. 2024 Feb 9;13:e50340. doi: 10.2196/50340 (PMC10891485; doi:10.2196/50340)
Supplement: Multimedia Appendix 1 [file resprot_v13i1e50340_app1.docx]

# Multimedia Appendix 1: Search strategy

PubMed (Date of last search: 8/8-2023)

| Search | Query | Records retrieved |
| --- | --- | --- |
| **Block 1 (Population - prediabetes)** | | |
| #1 | "diabetes mellitus, type 2/prevention and control"[MeSH Terms] OR "Prediabetic State"[MeSH Terms] | 16,979 |
| #2 | "prediabet*"[Text Word] OR "pre diabet*"[Text Word] OR "pre-DM"[Text Word] OR "impaired glucose toleran*"[Text Word] OR "impaired fasting glucose"[Text Word] OR "impaired fasting glycaemia"[Text Word] OR "impaired fasting glycemia"[Text Word] OR "intermediate hyperglycemia"[Text Word] OR "intermediate hyperglycaemia"[Text Word] OR "non diabetic hyperglycemia"[Text Word] OR "nondiabetic hyperglycemia"[Text Word] OR "non diabetic hyperglycaemia"[Text Word] | 29,515 |
| #3 | #1 OR #2 | 36,459 |
| **Block 2 (Intervention- digital interventions)** | | |
| #4 | "Digital Technology"[MeSH Terms] OR "Hotlines"[MeSH Terms] OR "Mobile Applications"[MeSH Terms] OR "Video Games"[MeSH Terms] OR "Virtual Reality"[MeSH Terms] OR "Wearable Electronic Devices"[MeSH Terms] OR "Telecommunications"[MeSH Terms] OR "Internet"[MeSH Terms] | 249,443 |
| #5 | "technolog*"[Text Word] OR "software*"[Text Word] OR "online"[Text Word] OR "internet"[Text Word] OR "virtual"[Text Word] OR "computer*"[Text Word] OR "digital*"[Text Word] OR "electronic*"[Text Word] OR "DVD"[Text Word] OR "DVDS"[Text Word] OR "video*"[Text Word] OR "television*"[Text Word] OR "tablet*"[Text Word] OR "telephone*"[Text Word] OR "phone*"[Text Word] OR "smartphone*"[Text Word] OR "mobile*"[Text Word] OR "hotline*"[Text Word] OR "telecommunication*"[Text Word] OR "tele communication*"[Text Word] OR "app"[Text Word] OR "apps"[Text Word] OR "text message*"[Text Word] OR "interactive voice response"[Text Word] OR "teleconsultation*"[Text Word] OR "tele consultation*"[Text Word] OR "short message service*"[Text Word] OR "sms"[Text Word] OR "email*"[Text Word] OR "e mail*"[Text Word] OR "telehealth*"[Text Word] OR "tele health*"[Text Word] OR "ehealth*"[Text Word] OR "e health*"[Text Word] OR "telemedicin*"[Text Word] OR "tele medicin*"[Text Word] OR "mhealth*"[Text Word] OR "m health*"[Text Word] OR "telenurs*"[Text Word] OR "tele nurs*"[Text Word] OR "wearable device*"[Text Word] OR "portable device*"[Text Word] OR "portable technolog*"[Text Word] OR "tracker*"[Text Word] OR "sensor*"[Text Word] OR "website*"[Text Word] OR "web site*"[Text Word] OR "web based*"[Text Word] | 3,521,814 |
| #6 | #4 OR #5 | 3,544,267 |
| **Block 3 (Publication type – randomized controlled trials)** | | |
| #7 | "Controlled Clinical Trial"[Publication Type] | 689,496 |
| #8 | "Controlled Clinical Trials as Topic"[MeSH Terms] | 172,849 |
| #9 | "rct"[Text Word] OR "placebo"[Text Word] OR (("random*"[Text Word] OR "controlled"[Text Word] OR "crossover"[Text Word] OR "cross over"[Text Word] OR "blind*"[Text Word] OR "mask*"[Text Word]) AND ("trial"[Text Word] OR "trials"[Text Word] OR "study"[Text Word] OR "studies"[Text Word] OR "analyz*"[Text Word] OR "analys*"[Text Word])) OR (("singl*"[Text Word] OR "doubl*"[Text Word] OR "tripl*"[Text Word]) AND ("blind"[Text Word] OR "mask"[Text Word])) | 2,184,866 |
| #10 | #7 OR #8 OR #9 | 2,186,272 |
| **Block 4 (Combined block searches)** | | |
| #11 | #3 AND #6 AND #10 | 825 |
| **Limited to** | | |
| #12 | #11 Filters: Danish; English; Norwegian; Swedish | 807 |
