# Supplementary material for: The Effectiveness of Digital Health Lifestyle Interventions on People With Prediabetes: Protocol for a Systematic Review, Meta-Analysis, and Meta-Regression
Source: JMIR Res Protoc. 2024 Feb 9;13:e50340. doi: 10.2196/50340 (PMC10891485; doi:10.2196/50340)
Supplement: Multimedia Appendix 2 [file resprot_v13i1e50340_app2.docx]

# Multimedia Appendix 2: Study selection process

**Identification of studies via other methods**

**Identification of studies via databases and registers**

Records identified from:

Websites (n = X)

Organisations (n = X)

Reference searching (n = X)

Citation searching (n = X)

Records removed *before screening*:

Duplicate records removed (n = X)

Records marked as ineligible by automation tools (n = X)

Records removed for other reasons (n = X)

Records identified from:

Databases (n = X)

Pubmed (n = X)

EMBASE (n = X)

CINAHL (n = X)

CENTRAL (n = X)

Registers (n = X)

**Identification**

Records excluded (n = X)

Records screened (n = X)

Reports not retrieved (n = X)

**Screening**

Reports sought for retrieval

(n = X)

Reports not retrieved (n = X)

Reports sought for retrieval

(n = X)

Reports excluded:

Wrong population (n = X)

Not digital intervention (n = X)

Wrong study design (n = X)

No subgroup analysis (n = X)

Not lifestyle intervention (n = X)

Digital component in control (n = X)

Wrong language (n = X)

Not effectiveness study (n =X)
Not adults (n = X)

Combined with pharmacological intervention (n = X)

Reports assessed for eligibility

(n = X)

Reports excluded:

Wrong population (n = X)

Not digital intervention (n = X)

Wrong study design (n = X)

No subgroup analysis (n = X)

Not lifestyle intervention (n = X)

Digital component in control (n = X)

Wrong language (n = X)

Not effectiveness study (n =X)
Not adults (n = X)

Combined with pharmacological intervention (n = X)

Reports assessed for eligibility

(n = X)

Studies included in review

(n = X)

Reports of included studies

(n = X)

**Included**
